# Recent Status of Nanomaterial Fabrication and Their Potential Applications in Neurological Disease Management

**DOI:** 10.1186/s11671-018-2638-7

**Published:** 2018-08-10

**Authors:** Khwaja Salahuddin Siddiqi, Azamal Husen, Sayed Sartaj Sohrab, Mensur Osman Yassin

**Affiliations:** 1 Department of Chemistry, Aligarh Muslim University, Aligarh, Uttar Pradesh 202002 India; 2 Department of Biology, College of Natural and Computational Sciences, University of Gondar, PO Box # 196, Gondar, Ethiopia; 3 Special Infectious Agents Unit, King Fahd Medical Research Center, King Abdulaziz University, PO Box # 80216, Jeddah, 21589 Saudi Arabia; 4 Department of Surgery, College of Medicine and Health Sciences, University of Gondar, PO Box # 196, Gondar, Ethiopia

**Keywords:** Nanomaterials/nanoparticles, Nanomedicines, Biomedical application, Drug delivery, Health and safety

## Abstract

Nanomaterials (NMs) are receiving remarkable attention due to their unique properties and structure. They vary from atoms and molecules along with those of bulk materials. They can be engineered to act as drug delivery vehicles to cross blood-brain barriers (BBBs) and utilized with better efficacy and safety to deliver specific molecules into targeted cells as compared to conventional system for neurological disorders. Depending on their properties, various metal chelators, gold nanoparticles (NPs), micelles, quantum dots, polymeric NPs, liposomes, solid lipid NPs, microparticles, carbon nanotubes, and fullerenes have been utilized for various purposes including the improvement of drug delivery system, treatment response assessment, diagnosis at early stage, and management of neurological disorder by using neuro-engineering. BBB regulates micro- and macromolecule penetration/movement, thus protecting it from many kinds of illness. This phenomenon also prevents drug delivery for the neurological disorders such as Alzheimer’s disease (AD), Parkinson’s disease (PD), multiple sclerosis, amyotrophic lateral sclerosis, and primary brain tumors. For some neurological disorders (AD and PD), the environmental pollution was considered as a major cause, as observed that metal and/or metal oxide from different sources are inhaled and get deposited in the lungs/brain. Old age, obesity, diabetes, and cardiovascular disease are other factors for rapid deterioration of human health and onset of AD. In addition, gene mutations have also been examined to cause the early onset familial forms of AD. AD leads to cognitive impairment and plaque deposits in the brain leading to neuronal cell death. Based on these facts and considerations, this review elucidates the importance of frequently used metal chelators, NMs and/or NPs. The present review also discusses the current status and future challenges in terms of their application in drug delivery for neurological disease management.

## Review

### Background

Nanomaterials (NMs) are expressed as materials having typical sizes in the range of 1–100 nm. Basically, they are composite-based (combine NMs with other NMs or with larger, bulk-type materials), dendrimer-based (nanosized polymers built from branched units), carbon-based (fullerenes, nanotubes), and metal-based (quantum dots, nanosilver, nanogold, and metal oxides viz. cerium oxide, titanium oxide, iron oxide, and zinc oxide) materials. In this cutting-edge century, fabrication of these nanoparticles (NPs), one by one or cluster, with desired particle size and shapes led to many promising applications in drug-gene delivery, disease management, pharmaceuticals, cosmetics, food, photonic crystals, coatings, paints, catalysis, bioremediation, material science, plant growth, and/or their production and protection [1–12].

The use of NMs at commercial and industrial levels has considerably increased, for instance about 3000 tons of TiO_2_ NPs per year was produced [13] and more than 50% was used in personal care products such as sunscreens [14]. Likewise, silver and gold NPs have been extensively used in medicine, disease diagnostic, sensor technology, biological leveling, pharmaceuticals, and many other biomedical applications [2, 11, 15–18]. Depending on their magnetic properties, iron and iron oxide NPs have been widely employed for cancer treatment, drug delivery, MRI, catalysis, and removal of pesticides from potable water system [11]. Platinum NPs are used as antioxidants and catalysts [10, 19], while palladium NPs are widely applied as catalysts and in cancer therapy [10].

In recent years, these NMs are being used as nanomedicines and play a vital role in diagnosis and treatment of numerous neurological disorders globally. Thus, nanomedicine is an emerging field where engineered NMs are utilized for the detection, treatment, and prevention of multiple diseases including neurological disorders. Nanomedicines are made up of nanoscale molecules with higher drug bioavailability. Often, NMs are designed to not interact with body defense mechanisms. The NMs are smaller in size and they can easily be stored into peripheral tissues for longer period availability in the body [20]. NMs can interact with physiological systems at the molecular and supra molecular level. They can be redesigned to respond against cell milieu and trigger desired biological activities in cell and tissue with reduced adverse effect. The novel nanotechnological inventions are making a valuable therapeutic contribution into the treatment and reduction of life-threatening diseases along with the neurological disorders [21].

Almost all neurological disorders are associated with the central and peripheral nervous systems. The brain, spinal cord, and nerves control the entire working of the body system. If anything goes incorrect with the nervous system, subsequently, problems related to speaking, swallowing, breathing, learning, etc. are commonly detected. The neurological disease treatment and management options are very limited because of the blood-brain barrier (BBB) which restricts the crossing and poor solubility of therapeutic molecules and desired drugs by the oral route. To overcome this issue, nanotechnology has provided an opportunity in novel technological inventions in the form of nanotubes, nanowires, nanospheres, robots, miniatures, nanosuspensions, nanomedicines, nanogels, nanoemulsions, nanocarriers, microparticles (MPs), NPs, polymeric and solid lipid NPs (SLNs), solid lipid carriers, liquid crystals (LCs), liposomes, microemulsions (MEs), and hydrogels for the effective and targeted drug delivery system and various disease diagnosis and management [22].

Currently, continuous efforts are being made by various research groups working on the neurological disorders in developing nanomedicines for targeted drug delivery by using NMs for the effective control and management of neurological disorders. Most frequently reported neurological disorders are Alzheimer’s disease (AD), Parkinson’s disease (PD), amyotrophic lateral sclerosis (ALS), multiple sclerosis (MS), neurological tumors, and ischemic stroke [23]. Among these, AD is categorized by loss of memory, loss of lexical access, and judgment impairment. It is an age-related disorder and increases with advancing age (60–85 years). Beside old age, obesity, diabetes, and cardiovascular disease are major factors for rapid deterioration of human health and on onset of AD. Mutations of genes have been described to cause the early onset familial forms of AD and they are known for coding amyloid precursor protein (APP) on chromosome 21 [24], presenilin 1 (PS1) on chromosome 14 [25], and presenilin 2 (PS2) on chromosome 1 [26]. The late onset sporadic form of AD embodies more than 90% of all diseases. The etiology of disease doubles each year after the age of 65 and reaches 50% at 85 years of age [27]. The genetic risk for the sporadic form of AD is due to inheritance of ε4 allele of apolipoprotein E which is located on chromosome 19q13 [27]. This protein can affect the progression of the disease and the extent of neurological cell damage [27, 28]. In view of this, numerous mechanisms have been postulated to elucidate the influence of apolipoprotein E in Alzheimer’s disease patients’ brain [28]. This protein also has a risk factor for the growth of mild cognitive impairment (MCI) which may later convert to AD development [29]. AD contributes in more than 80% of dementia and now it has been categorized as the most devastating disease in the world [20, 30–32]. Environmental pollution is the major cause of AD and PD progression. Metal and metal oxide from different sources are inhaled and get deposited in the lungs/brain. For instance, CeO_2_ and TiO_2_ have demonstrated accumulation in tissues after long-term exposure [33, 34]. It has been verified that TiO_2_ NPs induced PD-like symptoms in zebrafish larvae and PC 12 cell lines. It induced premature hatching and disturbed their locomotion [35]. The TiO_2_ NPs in the brain tissues of zebrafish have been shown to induce ROS generation leading to cell death in the hypothalamus region. These NPs also affect the neuron function. In a recent study, Yoo et al. [36] have demonstrated that gold NPs enable the generation of induced dopamine neurons for PD treatment in the presence of electromagnetic fields.

As mentioned, the bioavailability and effective delivery of drugs and other therapeutic compounds in the nervous system is restricted by two barriers namely BBB and blood cerebrospinal fluid barrier (BCSFB) [20, 37–40]. The BBB plays a significant role to protect the entry of blood-borne pathogens like bacteria, virus, parasites, and toxins [41]. Although the BBB facilitates a shield to the brain, it also interferes with the treatment of the numerous neurological disorders. It is therefore essential to develop a benign and effective drug delivery system which may cross the BBB and reach the target cells without producing any adverse effects. Vashist et al. [42] have reported that the BBB decreased concentration of drug that reaches the site of action and decreased its ability to treat the target disease; thus, higher concentration of drugs strengthened the need to develop nanomaterial-based drug delivery systems. The study also highlighted the recent trends of nanogel preparation and their significance in drug delivery system. It is important to note that either lipophilic molecules or low molecular weight molecules (below 400–600 Da) cross the BBB; thus, caution of drug selection is required for neurological disorder treatments. AD may be familial or sporadic, cognitive impairment, and plaque deposits in the brain leading to neuronal cell death. It is advisable to prevent the loss of functional neurons or to replace damaged neurons. Transplantation of neural stem cells (NSC) has been revealed to improve the cognition and synaptic conductivity in animal model of AD [43].

Zhang et al. [44] have reported the significance of NMs in stem cell therapy for several kinds of neurological diseases. The authors found that the NM promotes stem cell proliferation and differentiation both in vivo and in vitro, as well as contributes dominant roles in stem cell imaging and tracking. Trekker et al. [45] have also reported the significance of mesenchymal stem cells (MSCs) to treat ischemic stroke; however, their systematic delivery to the target remains a challenge. MSCs labeled with dextran-coated MNPs were disseminated in the brain to areas of enhanced cerebral lesion risk and revealed better functional recovery. The study reported that even though the intravenous administration routes were benign, the amount of MSCs that crossed the BBB was limited.

In this review, the main emphasis has been given on the frequently used metal chelators, NMs/NPs, and the current status in terms of their application in drug delivery system for neurological disease management.

### Neurological Disorders and Management

**Fig. 1:**
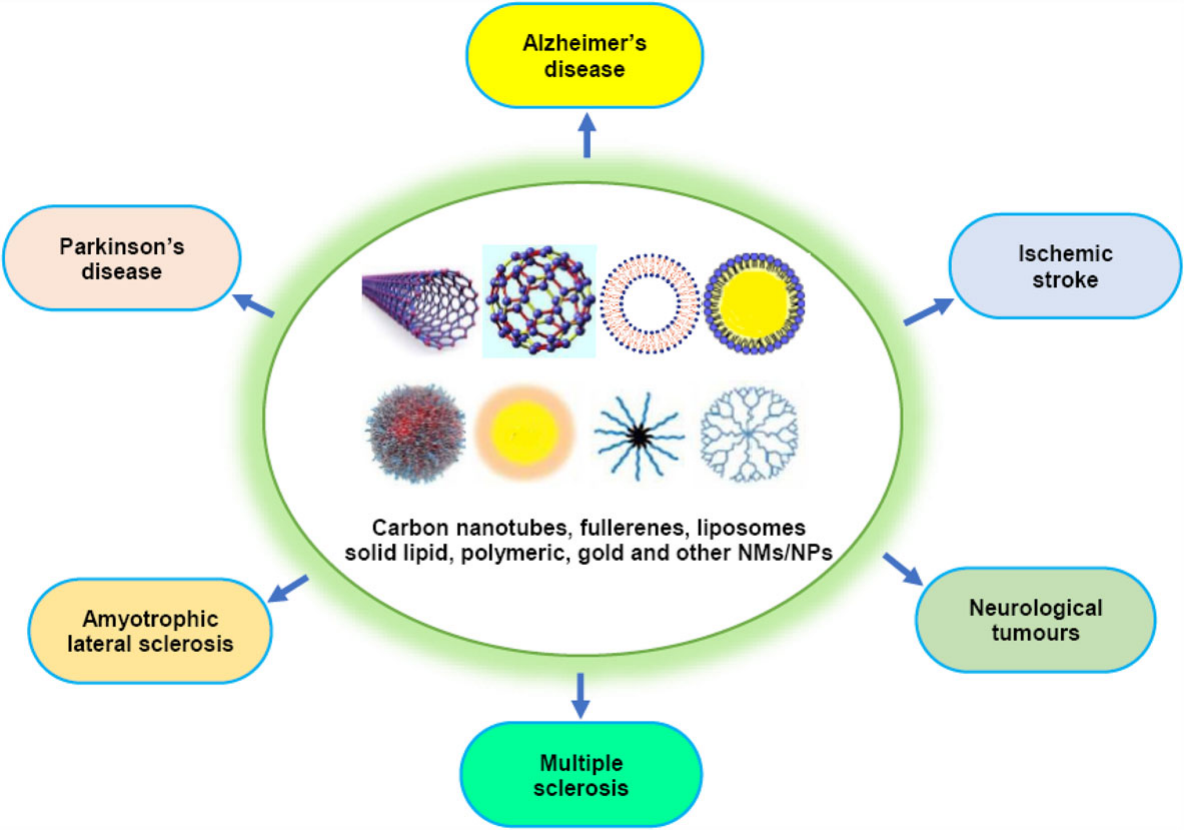
Different types of NPs and their application in neurological disorder treatment and management

Taken together, the CNS-associated main challenges are absence of smart diagnostic tools and incapability of effective drugs to cross BBB. To overcome these issues, various formulations of NMs/NPs have shown extensive and promising applications in drug delivery against neurological disorder treatment and management (Fig. 1). The specific application of NMs/NPs in neurological disorders like AD, PD, ALS, MS, neurological tumors, and ischemic stroke is given below.

#### Alzheimer’s Disease

Currently, AD has affected more than 35 million people and it is expected that by 2050 the cases will increase [22] at global level. At present, AD treatment is based on symptoms and vascular prevention by using cholinesterase inhibitors and *N*-methyl-D-aspartate antagonists. The use of nanotechnology in AD diagnosis and treatment has shown promising results. Multiple NMs are being used in AD diagnosis and treatment. Many methods have been utilized to prepare NPs such as polymer polymerization, ionic gelation emulsion, solvent evaporation, solvent diffusion, nanoprecipitation, spray drying, and particle replication in non-wetting templates. The condition of AD can be improved by using NPs which have a good affinity with the amyloid-β (Aβ) forms which induces “sink effect.” The diagnosis of AD and detection of Aβ1 has reached an advanced stage by using ultrasensitive NP-based bio-barcodes, immune sensors, and scanning microscopy procedures [46].

**Scheme 1:**
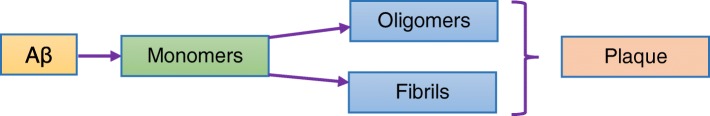
Plaque formation from amyloid-β protein

The main focus of the treatment is to target the metabolic dysfunction and aggregation of proteins and Aβ peptides. Plaque formation from Aβ protein is shown in Scheme 1 below:

The intracellular hyperphosphorylated neurofibrillary tangles and amyloid plaques (extracellular deposits of Aβ peptide) in the brain are the main cause of AD. Other reasons of AD progression have also been suggested, namely dysregulation of the cholinergic system and Aβ peptide deposition in the brain [31]. NFTs damage axonal integrity and neurotransmitter transport [47]. A drug should therefore be formulated with significant characters that can cross the BBB. The BBB protects the brain against variable pathogens. Lipophilic molecules, O_2_ and CO_2_, and other molecules with a molecular weight < 600 g/mol can easily diffuse across BBB. Amino acids, glucose, and insulin enter into the brain through specific receptor-mediated endocytosis [48]. Many devices have been developed by using multiple approaches in drug transportation to cross BBB and reach into the brain tissue of AD patients. One such approach is the conjugation of active compounds with nanocarriers viz. polymeric micelles, liposomes, lipid, and polymeric NPs having high association to BBB. Thus, the nanocarrier interaction with brain nutrient transport system allows the drug to reach the target site. For instance, Lockman et al. [49] have reported that the coating of NPs with thiamine is targeting the particles to BBB thiamine transporter. The drug is thus transported through BBB [50]. Biodegradable materials as a carrier are helpful in transporting the drug to the site of use. These treatments are expected to protect, repair, and regulate the damage in central nervous system (CNS) tissues [51].

The hydrophilic, charged, fluorescent marker ThT has been used as a probe for the detection of amyloid-β plaques of AD [52]. Hartig et al. [53] have delivered the encapsulated ThT NPs containing PBCA into the mice brain by intrahippocampal injection. In the study, TEM images have shown the presence of NPs in the microglia and neurons. The detection of AD can therefore be done by using this technique.

Biochemical investigation of the brain of AD patients has shown neocortical deficits in choline acetyltransferase [54] which is accountable for the acetylcholine (Ach) synthesis. It is also helpful in learning and sharpening of memory. It is therefore anticipated that generation of cholinergic neurons in the basal forebrain and loss of neurotransmission in the cerebral cortex contribute to the deterioration in cognitive function in patients suffering from AD. Treatment of rat with scopolamine, a ACh muscarinic receptor antagonist, reduced the levels of ACh with concurrent impairment of spatial memory [55]. However, it has been observed that substances which increase ACh release, viz. linopirdine, improve atropine-persuaded memory loss [56].

Polymeric NPs were made and encapsulated with radio-labeled 125I-clioquinol to enhance its transport to the brain and amyloidβ plaque retention of 125I-CQ. These NPs have been observed to be a suitable vehicle for in vivo single-photon emission computed tomography [22, 57]. Another NP known as magnetic iron oxide is being utilized efficiently as it has bigger surface area and magnetic effects with less toxicity. Gold NPs have been utilized as a valuable tool in kinetic studies for Aβ peptide aggregation. Additionally, heterodimeric NPs were synthesized by fusing gold NPs containing a cobalt(II) magnetic core and a platinum shell. These NPs were stabilized by coating with lipoic acid-PEG and showed promising result in AD [58]. Additionally, SLNs are typically spherical lipid core matrix which can efficiently solubilize lipophilic molecules. The SLNs can cross the BBB and drugs/therapeutic molecules could be efficiently delivered into the brain by endocytosis [22, 59].

Liposomes are another type of drug delivery vehicles and contain one or more phospholipid bilayers to carry lipophilic or hydrophilic drugs. The rivastigmine liposomes and cell-penetrating peptide-modified liposomes were formulated for improved distribution into the brain and reduced the side effect resulting into enhanced pharmacodynamics. The results showed that rivastigmine concentration across the BBB were higher after 8 h of delivery into the brain [60]. The surfactant-based drug delivery systems provided another option for drug delivery by aggregation of surfactant molecules in the presence of water to form structures based on the surfactant concentration, presence of salts, and temperature. The MEs are usually thermodynamically stable. Thus, microemulsion, nanoemulsions, and lyotropic LC mesophases can be generated with diverse geometries [22].

Two types of NPs such as polysorbate 80-coated poly (*n*-butyl cyanoacrylate) and another coated with polysorbate 80 were fabricated using emulsion polymerization to treat AD [61]. A dual functional NP was developed for delivery of drug based on PEGylated poly (lactic acid) polymer with two targeting peptides, TGN (a ligand composed of 12 amino acids: TGNYKALHPHNGC) and QSH (d D-enantiomeric peptide: QSHYRHISPAQVC) by conjugating at the surface of NPs and used in cases of AD [62]. TGN was used for targeting BBB ligands while QSH has effective association for Aβ plaques. These NPs were directly sent to Aβ plaques by targeted delivery in the brains of AD mice. Thus, it is expected that the use of NPs could be an important tool for AD diagnosis and treatment [22].

Postmortem studies of brain tissues from AD patients had indicated two types of lesions, namely senile plaques (SPs) and neurofibrillary tangles (NFTs). SPs in AD patient brain have been found to be augmented with copper, zinc, and iron. It is thought that the metals interact with metals and proteins which may influence aggregation of amyloid-β (Aβ) causing toxicity. Zinc, copper, and iron have been revealed from several clinical investigations, to be supplemented in Aβ plaques in transgenic mice [63–66]. Zinc and iron have been detected in NFT-containing neurons. Iron (III) and Cu (II) can chelate with proteins and alter their basic conformation promoting phosphorylation and aggregation. Metals have preference to bind with various atoms in proteins such as N, O, and S. Thus, metal chelates may be used in the treatment of AD and the excess metals in SPs may be removed by coordination with proteins. Aβ reduces copper (II) and iron (III) ions and produces H_2_O_2_ by double electron transfer to O_2_ [66].

$$ 2{\mathrm{H}}_2{\mathrm{O}}_2\to 2{\mathrm{H}}_2\mathrm{O}\kern0.5em +\kern0.5em {\mathrm{O}}_2 $$

This Aβ-induced oxidative stress and toxicity in cell culture is moderately arbitrated by methionine and tyrosine [67, 68]. Free radical-mediated reactions play a significant role in aging and physiology of many neurological diseases. Antioxidants such as polyphenolic compounds (resveratrol, curcumin, catechins) are found to be very helpful in AD treatment [69]. These compounds exhibit potent antioxidative and anti-inflammatory properties (Table 1), and numerous in vitro investigations have exhibited that green tea polyphenols could protect neuron from Aβ-induced damages [70–72]. Green tea polyphenols have exhibited positive influence in animal models of stroke/cerebral ischemia, AD, and PD. Green tea contains epigallocatechin gallate (EGCG) as an active ingredient that acts as a neuroprotectant against Aβ.

**Table 1:** The beneficial role of selected polyphenolic compounds in Alzheimer’s disease

Polyphenolic compounds	Target and role	Properties	Key references
Resveratrol	Aβ pathway	Remodels soluble oligomers and fibrils form into nontoxic form of Aβ	Ladiwala et al. [73]
	Aβ pathway	Reduction of production of Aβ peptides in vitro	Marambaud et al. [74]
	Cytoprotection	Protect cells from Aβ-induced toxicity	Han et al. [75]
	Oxidative markers	Decrease of ROS and lipid peroxide levels in animal models	Haque et al. [76]
	Synaptic density	Decrease of cognitive deficits in animal models	Kumar et al. [77]
	Specific proteins	Reduced the number of lysosomes and Aβ-induced toxicity	Regitz et al. [78]
	Mitophagy pathway	Reduced apoptosis, decreased oxidative status, and alleviated mitochondrial damage in Aβ_1–42_-treated PC12 cells	Wang et al. [79]
	Inhibiting the increase of protein kinase A and activation of PI3K/Akt signaling pathway	Alleviates Aβ_25–35_-induced dysfunction in hippocampal CA1 pyramidal neurons via recovery of the function of transient potassium channel and delay rectifier potassium channel by inhibiting the increase of protein kinase A and the activation of PI3K/Akt signaling pathway	Yin et al. [80]
Curcumin	Aβ pathway	Reduction of BACE-1 mRNA	Liu et al. [81]
	Aβ pathway	Reduction of the formation of Aβ fibrils	Ono et al. [82]
	Aβ pathway	Reduction of Aβ deposits and senile plaques in Tg2576 mice model	Yang et al. [83] Garcia-Alloza et al. [84] Lim et al. [85] Frautschy et al. [86]
	Cytoprotection	Protect cells from Aβ-induced toxicity	Kim et al. [87]
	Inflammatory pathways	Reduction of Aβ-induced expression of cytokines and chemokines	Lim et al. [85]
	Synaptic density	Increase of post-synaptic density-95 in vitro in the brain of Aβ-injected rats	Frautschy et al. [86]
	Cognitive deficits	Decrease of cognitive deficits in animal models	Frautschy et al. [86] Ishrat et al. [88]
Catechins	Aβ pathway	Reduction in the translation of APP mRNA	Levites et al. [71]
	Aβ pathway	Increase α-secretase activity; reduction in the production of Aβ peptides in APP695 over-expressing neurons	Rezai-Zadeh et al. [89]
	Aβ pathway	Reduction in β-secretase activity	Jeon et al. [90]
	Aβ pathway	Reduction in the formation of Aβ fibrils by binding to the native unfolded Aβ	Levites et al. [71] Ehrnhoefer et al. [91] Bieschke et al. [92]
	Cytoprotection	Protect cells from Aβ-induced toxicity	Levites et al. [71] Bieschke et al. [92]
	Cytoprotection	Reduction in Aβ-induced caspase activity in hippocampal neuronal cells	Choi et al. [70]
	Inflammatory pathways	Reduction in Aβ-induced cytokines in human astrocytoma U373MG cells	Kim et al. [93]
	Oxidative markers	Reduction in Aβ-induced levels of lipid oxidation in hippocampal neuronal cells	Choi et al. [70]
	Cognitive deficits	Decrease of cognitive deficits in animal models	Rezai-Zadeh et al. [89] Haque et al. [76]

Curcumin, an active component found in turmeric, works as a potent antioxidative and anti-inflammatory agent. When it was fed to aged Tg2576 mice, significant reduction of Aβ level and plaques was observed [83]. It also blocked Aβ aggregation and fibril formation in vitro (IC_50_ = 0.8 μM) which reduced amyloid plaques [83]. Curcumin possibly chelates the redox-active iron and copper [94]. Since its solubility in water is very low with rapid systemic elimination, low absorption, and degradation at alkaline pH, it is safe even at higher doses [95, 96]. Yang et al. [96] have reported that 10 mg kg^−1^ of curcumin given intravenously to rat yielded maximum serum curcumin level of 0.36 ± 0.05 μg ml^−1^, while a 50-fold higher oral curcumin dose gave only 0.06 ± 0.01 μg ml^−1^ serum level. However, Ravindranath and Chandrasekhara [97] have reported that the higher dose did not result in higher absorption. The drug molecules that are not ionized at physiological pH are lipophilic with low molecular mass and can cross BBB by diffusion. Neuropeptides, amino acids, and hexoses normally require a specific carrier to diffuse into the brain [98] although peptides and proteins can cross the BBB by saturable transport system [99].

Polymeric nanocarriers are promising candidates because they can open the tight junctions (Tjs) of BBB, prolong the drug release, and protect them against enzymatic degradation [41]. Hydrophilic NPs with less than 100 nm are very effective drug carriers. Bio-distribution increases with decreasing size of NPs. The distribution of the injected gold NP (15, 50, and 100 nm) in mice showed higher amount of NP with 15-nm particle size in the stomach, brain, heart, lung, liver, spleen, kidney, and blood. The larger particles are absorbed in a smaller amount in the stomach, pancreas, brain, and blood [100]. A number of factors are responsible for rapid transport of therapeutic drugs/molecules across BBB, for instance, molecular mass of drug, molecular charge, structural conformation, concentration gradient solubility, polymer used, and affinity of the drug to bind with certain donor sites/cellular proteins [101]. The nonappearance of toxicity at the BBB both in vitro and in situ suggests that the NPs may be transported via the barrier by endocytosis/transcytosis or even through diffusion. They may be taken up by brain endothelial cells [102]. It is, however, essential to examine the toxicity of the NP prior to its use as carrier. Drug-loaded NPs tested for the treatment of AD have been summarized in Table 2.

**Table 2:** Types of NPs and/or NMs for Alzheimer’s disease treatment

Type of NPs and/or NMs	Size	Drugs	Advantage and/or application	Key references
Polymeric NPs	1–1000 nm	Neuroprotective peptide, rivastigmine, curcumin, estradiol, S14G-humanin, anti Aβ antibody, fibroblast growth factor, Aβ-targeting peptide, iron chelator, selegiline, Aβ 1–15, ROCKII-siRNA, clioquinol	Drug-loaded NPs exhibited specificity for Aβ plaques both in vitro and in vivo; capable of aiding in the early diagnosis of Alzheimer’s disease	Hadavi and Poot [32] Sahni et al. [103] Gregori et al. [104] Wen et al. [105]
Liposomes, CPP-modified liposomes, flexible liposomes	200–500 μm	Curcumin, phosphatidic acid, cardiolipin, XO4, glycofused benzopyrane, anti Aβ antibody, ZnAc, EDTA, His, epigallocatechin-3-gallate, quercetin, rivastigmine HCl, galantamine	Beneficial for stabilizing therapeutic compounds, overcoming obstacles to cellular and tissue uptake, and improving bio-distribution of compounds to target sites in vivo. Present as an attractive delivery system due to their flexible physicochemical and biophysical properties, which allow easy manipulation to address different delivery considerations	Hadavi and Poot [32] Gregori et al. [104] Wen et al. [105] Sercombe et al. [106]
Solid lipid NPs and lipid-coated microbubble/NP-derived (LCM/ND)	50–1000 nm	Piperine, galantamine, lipoyl-memantine, rivastigmine HCl	Stabilizing drugs that suffer from physicochemical or biological instability; improving the bioavailability of drugs that cross the BBB; increasing permeating of drugs through the BBB	Wen et al. [105] Qu et al. [107] D’Arrigo [108]
Chitosan NPs	15–200 nm	Tacrine, Aβ fragment,	Enhanced concentration of drug in the brain, more stable, permeable, and bioactive	Sahni et al. [103] Gregori et al. [104] Wen et al. [105] Ahmed et al. [109]
Magnetite NPs	1 nm to 5 μm	Tacrine	Useful as selective biomarkers for detecting the location and the removal of other amyloid plaques derived from different amyloidogenic proteins	Sahni et al. [103] Gregori et al. [104] Busquets et al. [110] Sara Teller et al. [111] Chen et al. [112]
Albumin NPs	40–500 nm	Apo-E binding, tacrine	Enhanced brain uptake of NPs by cerebral endothelium, by an endocytic mechanism, followed by transcytosis into the brain parenchyma	Sahni et al. [103] Gregori et al. [104] Saraiva et al. [113] Karimi et al. [114]
Gold NPs	1–150 nm	Aβ-binding peptide	The prepared NPs dissolve toxic protein deposits of Aβ1–42 (amyloid deposits) by the combined use of weak microwave fields and gold NPs without any bulk heating	Hadavi and Poot [32] Sahni et al. [103] Gregori et al. [104] Gao et al. [115]
Exosomes	30–100 nm	BACE1-siRNA	Exosomes penetrate the blood-brain barrier and deliver drugs to the brain. They can be strategically engineered to carry drugs and possess a suitable half-life for many diseases	Gregori et al. [104] Sarko et al. [116] Quek et al. [117] Chen et al. [118] Jiang and Gao [119]
Polystyrene NPs	240 nm	Penicillamine	Deliver D-penicillamine to the brain for the prevention of Aβ accumulation	Hadavi and Poot [32] Sahni et al. [103] Saraiva et al. [113]
Core–shell NPs	–	Thioflavin T and S	Tools to trace and clear Aβ in the brain	Sahni et al. [103] Busquets et al. [110] Sonmez et al. [120]
Nanolipidic and microparticles	30–80 nm	Polyphenol EGCG, donepezil	Prevent Aβ formation. Acetylcholine esterase inhibitor with high specificity for acetylcholine esterase in the central nervous system	Hadavi and Poot [32] Sahni et al. [103]
Trimethylated chitosan conjugated-PLGA NPs	94 ± 8.1 to 146.5 ± 5.1	Coenzyme Q10(Co-Q10)	Q10-loaded TMC/PLGA–NP greatly improved memory impairment and restoring it to a normal level	Sahni et al. [103]
Poly(butyl) cyanoacrylate NPs	178 ± 0.59 to 197 ± 2.3	Apo-E binding	Attachment of ApoE3 to C-PBCA NPs increased the uptake of curcumin into cells as compared to the plain solution or untargeted NPs	Sahni et al. [103]
Nanoemulsions	10–1000 nm	Nano-PSO, lipid-coated microbubble/NP-derived (LCM/ND)-scavenger receptor class B type I	Good solubilization and protection of lipophilic drugs in the oil droplets and easy for large-scale production	Wen et al. [105] Mizrahi et al. [121]
Microemulsions	1–100 nm	Huperzine A and ligustrazine phosphate	Microemulsions are optically isotropic and thermodynamically stable liquid solution and showed great improvements in the cerebral cholinergic function and oxidative systems that further slow down the progression of Alzheimer’s disease	Wen et al. [105] Shi et al. [122]
Dendrimers	–	ADDL—amyloid-beta-derived diffusible ligands, (PPIG4-Mal) and fifth (PPI-G5-Mal) phosphorus-containing dendrimers	To modulate amyloidogenesis and stop the aggregation of Tau protein. Interfering with Aβ fibrilization in Alzheimer’s disease	Wen et al. [105] Tomasz [123]

None of the non-steroidal-based drugs namely phenserine, statins, tarenflurbil, tramiprosate, and xaliproden have exhibited satisfactory efficiency in treatment of neurological disorders [124–126]. However, it is known that high levels of cholesterol are related with increased risk of AD. It has been verified based on animal studies that hypercholesterolemia promotes Aβ production and deposition. Currently, there are also two classes of medication approved for the AD treatment. The choline esterase inhibitor (ChEI) donepezil (Aricept), galantamine (Reminyl), and rivastigmine (Exelon) are prescribed for the treatment of mild to moderate AD. The *N*-methyl-D-aspartate antagonist memantine is the only medicine for the treatment of moderate to severe dementia. Excess ions of iron, zinc, and copper cause precipitation of Aβ leading to the development of toxic Aβ oligomers [127]. Formation of Aβ oligomers can be easily prevented if the above metal ions are chelated with non-toxic ligands such as diferrioximme or D-penicillamine, giving soluble complexes which can be removed from living system. Polystyrene NPs of 240 nm conjugated with deferiprone administrated to cultured human cortical neurons in vitro showed decreased cytotoxicity by preventing Aβ aggregation [128]. However, the bioavailability and toxicity limit their application in the human system. Nanocarriers facilitate this property by conjugation of chelating agent with them.

Likewise, 5-chloro-7-iodo-8-hydroxyquinoline (a quinol derivative) is known to have high affinity for zinc and copper ions. Treatment of AD transgenic mice with this quinol blocked Aβ aggregation [129]. Soluble complex formulation in low concentration prevents the interaction of metal with other ligating proteins. The efficiency and bioavailability of quinol can be increased by encapsulating with PBCA NPs coated with polysorbate 80. These quinol NPs were reported to cross the BBB in wild-type mice indicating potential for the AD treatment [129].

**Scheme 2:**
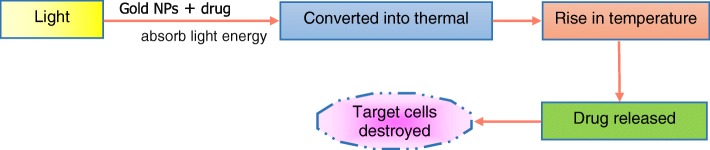
Role of gold nanoparticles in Alzheimer's disease treatment

Naturally occurring molecules have also been suggested in AD treatment. For instance, curcumin from turmeric and quercetin flavonoid from fruits and vegetables are anti-inflammatory, antioxidant, and anti-cancer in nature. Liposomes of 170 nm prepared from curcumin–phospholipid conjugates have demonstrated to have high affinity with Aβ fibrils in vitro and very low affinity to Aβ monomers [130]. Liposomes work as a carrier to deliver therapeutic molecules in AD patients. Similarly, quercetin has also demonstrated to protect primary rat hippocampal neurons from Aβ cytotoxicity, protein oxidation, lipid peroxidation, and apoptosis [131]. Oral doses of quercetin in mouse showed improvement in learning and memory capability but its absorption in intestine is low and causing its rapid elimination [132]. When liposome-encapsulated quercetin was nasally administered, it inhibited the degeneration of hippocampal neurons in rat model of AD [133]. The confirmation of the protein in AD plays a significant role. The peptide may adopt a β-sheet confirmation or coil formation. An appreciable decrease in insoluble and soluble Aβ peptide in mice brain has been observed. However, conformational change is significant in the treatment of AD. The gold NPs are frequently used in the treatment of AD under electromagnetic field. As shown in Scheme 2 below; the NPs loaded with drug are photothermally excised and absorb the light energy which is converted to thermal energy and increases the temperature of the NP which destroys the target cells without damaging the normal healthy cells.

#### Parkinson’s Disease

PD is a neurodegenerative disease that annually affects one individual in every 100 persons aged above 65 years. This disease causes severe complications in patient body motions by affecting neuro-inflammatory responses. The use of nanotechnology could be a powerful tool to alleviate PD. Engineered NMs can promote regeneration and protection of affected neurons and also enhance the drug and small molecule delivery across the BBB. To overcome the side effects of conventional therapy for PD, extensive research is currently being conducted on the development of many strategies and techniques like nano-enabled scaffold device for biometric simulation and optimization and direct and targeted delivery into the brain. Currently, peptides and peptide NPs are being used not only in PD but also in other CNS disease diagnosis and treatment. But further development with improved and effective performance is urgently needed for delivery of nanomedicines into the CNS and brain tissue [46]. Gold- and TiO_2_-incorporated nanotube arrays recognize a-syn using photoelectrochemical immune sensors [134]. AFM studies in tandem with nanoneurotechnology can recognize protein misfolding of single a-syn molecules. Neuroinflammation and neurodegeneration inside neurons is effectively reduced by using catalase-packaged polyethyleneimine NPs. Additionally, anti-α-syn-conjugated polybutylcyanoacrylate NPs helped in neuronal a-syn clearance [23, 135, 136].

#### Amyotrophic Lateral Sclerosis

It is a motor neuronal disease and causes the loss of neuromuscular control with fatal outcomes [137]. The degeneration of motor neuron occurs in both lower and upper neurons. Protein inclusions as well as superoxide dismutase 1 (SOD1) are predominantly detected in both neurons and axons. A SOD-coated gold NP combined with SOD1 aggregates can be used as colorimetric detection system for ALS diagnosis [138]. The neuroprotective pathology can be achieved by using carboxyfullerene nanotubes with SOD [139]. The effective and accurate delivery of riluzole, a glutamate inhibitor to the effected sites, can be performed by using carbon NPs [140, 141].

#### Multiple Sclerosis

MS is an often disabling CNS disease. The most common symptom is disruption of information flow to the brain and in between the brain and body. The disease progression and myeloid neuronal infiltration can be achieved by using a water-soluble fullerene unified with an *N*-methyl-D-aspartate receptor antagonist in diseased patients tested poly (methyl methacrylate) and poly (caprolactone)-PEG (PCL-PEG) NPs. The therapeutic effect of the drug was increased in mice [142–144]. In another study, the co-polymers of PEG were used to load cells with catalase and finally it was delivered intravenously and the therapeutic activity was observed to increase in the inflamed brains [145]. Additionally, the disease severity was reduced by using poly (ethyleneimine) loaded with a therapeutic DNA in mice [58, 146].

#### Neurological Tumors

The treatment of neurological tumors (like brain tumors) has been investigated for many years by using polymeric NPs [147]. For the treatment of most of the tumor, a passive targeting technology using smaller than 100-nm NPs has been used with enhanced permeability, penetration, and retention effect which resulted into better gathering of NPs around the tumor region [143, 144]. The risk of elimination of NP, targeting brain tumor from the blood, can be overcome by engineering the better surface with receptors like folate which facilitate the NP accumulation at their site of action [142, 143]. Cabral and Kataoka [144] have suggested that the use of polymeric NPs for brain tumor study has reached an advanced stage of pre-clinical phase. The BBB was disrupted in many brain tumors except micrometastases or infiltrative gliomas [148]. Paclitaxel-loaded PEGylated PLGA-based NP was designed to target brain gliomas, and it was observed that the life span of mice increased twofolds [58, 145].

#### Ischemic Stroke

Currently, at global level, ischemic stroke is considered as a third root cause of death. It produces structural brain damage. The targeted and effective delivery of drugs and therapeutic compounds in the brain can be achieved by using stereotactic surgery [146]. Ischemic stroke treatment using nanomedicine in the brain has been already demonstrated [149]. CNTs are found to be very useful in brain imaging to identify stroke location and diseased site as well as delivery of drugs/therapeutic molecules to the site of action. The drug delivery by using nanotechnology will be a valuable tool for ischemic stroke and other chronic neurological diseases. Single-walled carbon nanotubes (SWCNTs) functionalized with amine groups increased the neuron tolerance to ischemic injury [147]*.* Application of nanodrug delivery could be of great benefit in the future for neuroprotection success in chronic neurological diseases including ischemic stroke. Neurotherapy with the use of CNTs would be extremely useful in the treatment of various neurological pathologies including ischemic stroke. Neurotrophin plays a significant role in the development and function of neurons as well as neuroprotection in both CNS and peripheral nervous system, and their delivery into the brain can be performed by using CNTs. The neuronal injury can be protected and functional motor recovery will be enhanced by pre-treatment with amine, functionalized with SWCNTs [20, 150].

### Metal Chelators and NMs/NPs Used in Neurological Disease Management

#### Metal Chelators

Metal chelators or multidentate organic molecules form complexes with metal and are more stable than those formed with monodentate ligands. If these complexes are soluble in aqueous medium, they can easily be removed from the biological system and prevent toxicity. There are several such molecules such as desferrioxamine, an iron chelator, but it has also been used in the depletion of zinc, copper, and aluminum [151] in AD patients. Penicillamine is specifically used for the removal of copper from the brain. Although many transition metals are essential to human subjects in trace amounts, they become toxic when they exceed the tolerance limit and are involved in neuronal damage in neurological diseases. For instance, enhanced quantity of copper (390 μM), zinc (1055 μM), and iron (940 μM) has been observed to be present in AD brain in comparison to the normal adult samples (copper 70 μM, zinc 350 μM, and iron 340 μM) [63, 152, 153].

#### Nanomaterials

Currently, NMs are being frequently used in tissue engineering and targeted drug delivery. They play a significant role to overcome major problems related to effective and targeted drug delivery into the brain for diagnosis and treatment of neurological disorders [154, 155]. BBB allows free diffusion and transport of lipophilic molecules, oxygen, and carbon dioxide, and transporters or receptor-mediated endocytosis help the entry of other compounds in the brain [48]. Thus, to overcome these barriers and improve the effective delivery of therapeutic compounds in the brain, now, multiple tactics are being used viz. nanocarriers and strong conjugation of valuable drug compounds to the vectors having active transport capacity of drugs through BBB in the brain. Several NMs are produced using nanotechnology that can deliver desirable therapeutic compounds into the brain tissues as well as near the site of drug action in other tissues [32, 50, 51, 156]. Biodegradable materials as a carrier also revealed an effective drug delivery near the site of action. Thus, these preparation and treatments are likely to protect, repair, and regulate the damage of CNS tissues [51]. In addition, many NMs and polymers are extensively being used in the drug delivery system by coating with surfactant polysorbate 80 enabling them to easily cross BBB through receptor-mediated endocytosis. These polymers are known as polylactic acid, polyglycolic acid, polylactic-co-glycolic acid, polycaprolactone, chitosan, gelatin, and polybutyl cyanoacrylate [39, 154]. These NMs have additional properties as their surface can be manipulated and or engineered with hydrophilic polyethylene glycol layer allowing to protect the drugs from enzymatic degradation and recognition by the immune system [157]. Thus, these significant features enable those compounds to be considered as promising vehicle for AD and other neurological disease diagnosis and treatment [32].

#### Polymeric Nanoparticles

Polymeric NPs are solid colloidal particles containing macromolecular materials to attach, adsorb, dissolve, and encapsulate the drugs or therapeutic compounds. Degradable polymeric NPs of 10–100 nm are a common type of drug delivery systems for the neurological disease treatments. These particles exist in two variable units, nanocapsules and nanospheres [58, 148, 158–160]. Nanocapsules are made of coreshell NPs, whereas nanospheres contain homogeneous matrices. These particles sizes facilitate fine tuning to acquire desired properties like active compound protection with easy delivery and permeability of drugs into the target cells with higher efficacy and efficiency at low cost preparation [161–163]. Moreover, these particles are effective due to suitable degradation rate and their capability to cross BBB and reach the CNS [154]. Coating of suitable polymer with surfactant polysorbate 80 enables them to cross the BBB by adsorption of apolipoprotein E from the blood which is taken up by the cells of BBB by endocytosis [154]. Some modification in the characteristic preparation of NP coated with polymers may occur which protects the drug against immune system/enzymatic degradation [157]. Different signaling pathways are activated when interaction of growth factors (GFs) with their receptors on cell surface occurs. All pathways are different from each other. From animal studies, it has been observed that insulin-like growth factor (IGF), basic fibroblast growth factor (bFGF), and nerve growth factor (NGF) available in the brain exhibit useful influences [155]. It is, however, difficult to deliver GFs due to BBB, enzymatic degradation, clearance, and denaturation in the brain and the blood [164]. Kurakhmaeva et al. [165] revealed from animal studies that NGF-loaded poly (butyl cyanoacrylate) (PBCA) coated with polysorbate 80 improved memory function in mouse model. Intravenous administration of drug is an alternative route of transportation to the brain. It is expected that the drugs/therapeutic molecules are taken up by the olfactory epithelium and transported to the cerebrospinal fluid by passing the BBB [166]. Polymer NP of 120 nm loaded with the bFGF coated with *Solanum tuberosum* lectin has been shown to improve learning and memory capability in rat model of AD [167]. In addition, many polymeric NPs have been designed to treat brain tumors and neurodegenerative disorders [58]. They may be encapsulated as therapeutic agent and transported into the brain if it crosses the BBB.

#### Solid Lipid Nanoparticles

SLNs are also being used as efficient and alternative carriers for drug delivery as they have better advantages with improved characteristics. SLNs are known as an attractive colloidal drug carrier system for brain targeting. The accumulation of SLNs in reticulo endothelial system limits their use for targeted drug delivery in the brain. The lipid matrix is solid at room temperature with unique size and their better advantages to use as nanocarriers which allows better release and stability of drugs without causing cytotoxic effects in the tissue [41]. The SLNs have better advantages of reproducibility by using multiple strategies and larger scale-up feasibility. It is also a good option for other formulations that lack organic solvents. This also reduces the chance of residual contaminations. Based on these characters, SLN provides one of the most promising systems for drug delivery against many neurodegenerative disease and cancer treatment [40, 168, 169]. The drug stability into the blood and their entry through BBB can be enhanced by using NMs with SLN formulations as the polysorbate triggers the serum proteins by acting as anchor for apolipoproteins. The NPs coated with polysorbate provided desirable results for effective delivery of drugs across the BBB. The interaction of lipoproteins with capillary endothelial cell receptors available in brain with apolipoproteins facilitates the crossing of BBB. The phagocytosis can also be prevented by surface modification of SLN by coating with hydrophilic polymers or surfactants [170]. Furthermore, the use of ligands to SLN surface also improves the drug concentration and increased drug stability and availability across BBB for the neurological treatments. However, to date, only few drugs are FDA-approved for AD, known as acetylcholinesterase inhibitors (donepezil, galantamine, and rivastigmine). Nonetheless, recently, solid NPs having galantamine hydrobromide have been developed to upgrade the drug bioavailability for AD treatment [40, 171].

#### Liposomes

Liposomes are spherical vesicles made of impermeable lipid bilayer, phospholipids, and cholesterol. They are being considered as an important vehicle for drug delivery due to their non-toxic and biocompatibility characteristics. They can deliver hydrophilic and hydrophobic molecules by carrying the aqueous and lipid parts of the liposomes. Though, they are recognized as foreign particles by the biological system without causing any negative response after their entry into the system, they are non-immunogenic as well as non-carcinogenic, biodegradable, and non-thrombogenic in nature [172]. Liposomes are being used as larger transport nanocarriers as they are capable of encapsulating multiple components. Additionally, they are protected against enzymatic degradation and removal by the reticuloendothelial system. The most important characteristics are capability to fuse with biological membranes, move across cell membrane, and to penetrate the BBB. The half-life of liposome can be easily enhanced by treating their surface with PEG [173]. The Aβ oligomers with high affinity towards liposomes can be used for delivery of therapeutic compounds in animal models [174]. In an in vitro study, using phosphatidylcholine liposomes having omega-3 fatty acid and docosahexaenoic acid into APP-overexpressing cells, it was observed that the cell membrane fluidity increased. The induction of non-amyloidogenic processing of APP resulted into formation of soluble APPα (sAPPα) and further the inhibition of JNK stress signaling pathway by sAPPα-containing cell supernatants; PI3K/Akt survival pathway was activated in cultured neuronal cells and finally resulted into prevention of apoptotic cell death [175]. So, liposomes containing DHA could be used for prevention and treatment of AD [32].

#### Gold Nanoparticles

Gold NPs are being effectively utilized for drug delivery against various diseases [17]. They have many important characteristics such as better biocompatibility, easy synthesis, and simplistic surface functionalization with easy and effective delivery to target cells and tissues [17, 18]. Some reports have shown that the gold NPs can be utilized in AD disease treatment by destructing and dissolving the Aβ fibrils and plaques with the help of weak microwave field exposure in the brain tissue. Major cases of AD are plaque formation and Aβ fibrils in the brain which can be either prevented or destroyed. Gold NP interaction with fibrils followed by their exposure to weak microwaves causes an increase in the temperature and dissolution of fibrils. Experiment in mice (in vitro) has shown that gold NPs slow down the progression of AD. It is also interesting to note that apparently NPs do not adversely affect the brain [176]. Gold NPs conjugated with some compounds interfering with Aβ fibrils have been used [114, 115]. Gao et al. [115] have reported that the gold NPs of 22-nm size reduces the cytotoxicity of Aβ fibrils and Aβ-mediated peroxidase activity in vitro. Triulzi et al. [177] have demonstrated the photochemical ablation of Aβ plaques in AD. They have suggested that gold NPs formed complexes with synthesized β-amyloid peptides. Upon irradiation with laser beam, the complex containing NP was stabilized. Gold NP conjugated with ematoporphyrin has been reported to be effective against T cell lines MT-4 and Jurkat cells (human T cell leukemia) [178] in vitro. They have been used as probe to detect neuronal cell activity [148]. Gold NP suspension of drug from nanobubbles can deliver the drug to the target site when the bubble bursts by heating. Based on these results, the use of gold NPs is a better option in AD disease diagnosis, treatment, and management [32, 115]. Overall, the metal NPs have shown a considerable potential in the treatment of neurological diseases.

#### Microparticles

MPs are basically a heterogenous population of small cell-derived (0.1–1 μm) vesicles and are now being used as an important vehicle for drug delivery and AD treatment. In the CNS, these particles have been detected in the CSF, where they are discharged by almost all types of cells [179, 180]. It is well known that the FDA-approved donepezil drug is being used in the improvement of daily life functioning and cognition of mild-to-moderate AD patients without causing any damage and significant changes in the function of vital organs till > 98 weeks. This medicine is being used as a daily dose but it causes gastrointestinal side effects as well as impaired memory. Nonetheless, this problem could be solved now by using PLGA donepezil-loaded microparticles for long-term use [181]. These particles were implanted subcutaneously in rats which resulted in steady-state plasma levels of donepezil for 4 weeks, and then, this drug was rapidly reduced. In another study, microparticles were used on rat after ligating with common carotid arteries and neuronal loss with reduced learning and memory capabilities was reported. The above result indicates that the use of FDA-approved drugs can be more beneficial with control release strategies for the treatment of AD [32, 182].

#### Carbon Nanotubes and Fullerenes

The carbon nanotube (CNT) was discovered in 1991 by Iijima [183]. They have many valuable properties such as ultra-light weight, high flexibility, low deposition, low cost, high capability, ultra-strong, and inert with electrical and thermal conductivity. Currently, it has emerged as new promising NMs due to useful and exclusive properties for treatment of neurological disorders viz. in AD, PD, and ischemic stroke [20, 184, 185]. The successful utilization of CNTs as drug delivery vehicles in vivo has been reported in many diseases like bone implants, rheumatoid arthritis, osteoporosis, and cancer [184, 186]*.* However, very limited preclinical studies have been performed for successful application of CNTs in neurological disorders [187]. Fullerene derivatives have also been investigated for their role as neuroprotective agents [188]. For instance, nanostructures of hydrated C60 fullerene (C60HyFn) showed protection on the CNS in rats against chronic alcoholization [189]. Authors have suggested an indirect participation of C60HyFn in the neurotransmitter metabolism. In addition, some reports have also shown that the fullerene derivatives contain multiple synergistic mechanisms that can be employed for AD treatment [190].

## Conclusions

All neurological disorders are associated with the spinal cord and nervous system. AD leads to the cognitive impairment and plaque deposits in the brain leading to neuronal cell death. Hence, it has been suggested to prevent the loss of functional neurons or to replace the damaged neurons. BBB provides protection to the brain, so an important challenge for any drug is to cross the BBB and to reach the CNS with desirable amount. It is therefore crucial to develop a benign and effective drug delivery system with improved efficacy which may effectively cross the BBB and reach the target cells without producing any significant adverse effects. Different NMs and/or NPs have been developed, utilized, and tested and showed promising contribution in the diagnosis, treatment, and management of neurological disorders. Drug-loaded NPs are tested for AD treatment and provided promising results. In addition, the significance of NMs in stem cell therapy for several kinds of neurological diseases is elucidated. NMs are also able to promote stem cell proliferation and differentiation and also contribute dominant roles in stem cell imaging and tracking. Thus, in CNS-related diseases, the use of NMs/NPs in drug delivery is a better option in comparison to the conventional mode of treatments. However, their systematic toxicity investigations are also required for the effective formulation and application in neurological disorders.

## Abbreviations

AD: Alzheimer’s disease
ALS: Amyotrophic lateral sclerosis
APP: Amyloid precursor protein
Aβ: Amyloid-β
BBB: Blood-brain barrier
CNS: Central nervous system
CNT: Carbon nanotube
DHA: Docosahexaenoic acid
FDA: Food and Drug Administration
LCs: Liquid crystals
MEs: Microemulsions
MS: Multiple sclerosis
MPs: Microparticles
MSCs: Mesenchymal stem cells
NFT: Neurofibrillary tangles
NMs: Nanomaterials
NPs: Nanoparticles
PBCA: Poly (butyl cyanoacrylate)
PD: Parkinson’s disease
PLGA: Poly(d,l-lactic-co-glycolic acid)
ROS: Reactive oxygen species
SLN: Solid lipid nanoparticle
SOD: Superoxide dismutase
SWCNTs: Single-walled carbon nanotubes
